# Supplementary material for: Effectiveness of the second COVID-19 booster against Omicron: a large-scale cohort study in Chile
Source: Nat Commun. 2023 Oct 27;14:6836. doi: 10.1038/s41467-023-41942-y (PMC10603055; doi:10.1038/s41467-023-41942-y)
Supplement: Supplementary file 1 — Supplementary Information [file 41467_2023_41942_MOESM1_ESM.pdf]

Supplementary Information

Effectiveness of the Second COVID-19 Booster Against Omicron: A Large-Scale Cohort Study in Chile

Alejandro Jara,[1,2] Cristobal Cuadrado,[3,4] Eduardo A. Undurraga,[5,6,7,8] Christian García,[3] Manuel Nájera,[3] María Paz Bertoglia,[3] Verónica Vergara,[3] Jorge Fernández,[3] Heriberto Garcia,[3] and Rafael Araos,[6,9,10*]

[1] *Facultad de Matemáticas, Pontificia Universidad Católica de Chile, Santiago, Chile*

[2] *Center for the Discovery of Structures in Complex Data (MiDaS), Santiago, Chile*

[3] *Ministerio de Salud de Chile, Santiago, Chile*

[4] *School of Public Health, Universidad de Chile*

[5] *Escuela de Gobierno, Pontificia Universidad Católica de Chile, Santiago, RM, Chile*

[6] *Multidisciplinary Initiative for Collaborative Research in Bacterial Resistance (MICROB-R), Santiago, Chile*

[7] *Research Center for Integrated Disaster Risk Management (CIGIDEN), Santiago, Chile*

[8] *CIFAR Azrieli Global Scholars program, CIFAR, Toronto, Canada*

[9] *Instituto de Ciencias e Innovación en Medicina, Facultad de Medicina, Universidad del Desarrollo, Santiago, Chile*

[10] *Advanced Center for Chronic Diseases (ACCDiS), Santiago, Chile*

[*] Correspondence to Dr. Rafael Araos at Instituto de Ciencias e Innovación en Medicina, Facultad de Medicina Clínica Alemana Universidad del Desarrollo, Av. Las Condes 12461, Las Condes, Región Metropolitana, Chile. rafaelaraos@udd.cl

Supplementary Table S1. Characteristics of the study cohort of adults aged 20 years or older affiliated to FONASA, with laboratory-confirmed COVID-19, unvaccinated and vaccinated individuals who received three (booster) or four doses (second booster) of COVID-19 vaccines, August 11, 2021, through August 15, 2022*

							Primary vaccination series + booster dose			
			COVID-19		Unvaccinated		First booster		Second booster	
Characteristic	No.	Col.%	No.	Row%	No.	Row%	No.	Row%	No.	Row%
Total	3,754,785	100.0	401,121	10.7	757,726	20.2	305,861	8.1	2,623,802	69.9
**Sex**										
Female	2,188,038	58.0	259,505	12.0	361,108	16.5	179,354	8.2	1,608,708	73.5
Male	1,566,747	42.0	141,616	9.0	396,618	25.3	126,507	8.1	1,015,094	64.8
**Region of residence**										
Arica	45,796	1.2	6,809	15.0	12,203	26.7	3,019	6.6	29,852	65.2
Tarapacá	62,515	1.7	5,650	9.0	18,168	29.1	4,355	7.0	39,130	62.6
Antofagasta	99,523	2.7	7,718	7.8	25,468	25.6	6,653	6.7	66,037	66.4
Atacama	59,408	1.6	7,268	12.0	11,565	19.5	4,583	7.7	42,367	71.3
Coquimbo	173,457	4.6	18,978	11.0	32,102	18.5	13,630	7.9	124,606	71.8
Valparaíso	434,271	12.0	46,028	11.0	93,877	21.6	35,777	8.2	296,251	68.2
Metropolitana	1,367,308	36.0	129,822	9.5	304,314	22.3	96,159	7.0	946,353	69.2
LB O'Higgins	210,852	5.6	19,187	9.1	32,769	15.5	17,256	8.2	156,822	74.4
Maule	249,203	6.6	33,636	13.0	39,533	15.9	21,612	8.7	183,284	73.6
Ñuble	129,216	3.4	17,942	14.0	16,588	12.8	11,742	9.1	98,463	76.2
Biobío	359,018	9.6	41,534	12.0	52,439	14.6	30,831	8.6	269,623	75.1
Araucanía	230,569	6.1	29,028	13.0	47,930	20.8	24,138	10.5	152,587	66.2
Los Ríos	85,879	2.3	10,760	13.0	17,231	20.1	8,484	9.9	58,464	68.1
Los Lagos	191,621	5.1	19,011	9.9	42,400	22.1	22,963	12.0	120,503	62.9
Aysén	20,407	0.5	3,020	15.0	4,100	20.1	2,002	9.8	13,970	68.5
Magallanes	35,742	1.0	4,730	13.0	7,039	19.7	2,657	7.4	25,490	71.3
**Age group†**										
20-29	290,877	7.7	43,471	15.0	147,632	50.8	16,574	5.7	124,462	42.8
30-39	378,950	10.0	57,451	15.0	175,679	46.4	21,991	5.8	178,109	47.0
40-49	362,335	9.6	49,520	14.0	123,358	34.1	20,316	5.6	216,088	59.6
50-59	572,417	15.0	67,016	12.0	103,081	18.0	35,526	6.2	429,326	75.0
60-69	1,051,931	28.0	95,104	9.0	76,780	7.3	80,732	7.7	880,181	83.7
70-79	724,506	19.0	57,528	7.9	51,925	7.2	68,026	9.4	587,503	81.1
80 or more	336,301	9.0	28,191	8.4	47,756	14.2	61,715	18.4	203,279	60.5
**Comorbidities**										
None	1,919,374	51.0	201,901	11.0	600,004	31.3	126,474	6.6	1,168,596	60.9
≥ 1	1,835,411	49.0	199,220	11.0	157,722	8.6	179,387	9.8	1,455,206	79.3
**Nationality**										
Chilean	3,548,923	95.0	392,654	11.0	605,160	17.1	299,611	8.4	2,579,106	72.7
Non-Chilean	205,862	5.5	8,467	4.1	152,566	74.1	6,250	3.0	44,696	21.7

**Notes. ***COVID-19 denotes coronavirus disease 2019. †The analysis uses age in years; the table includes age groups to show the age distribution in the cohort. The Ministry of Health launched a COVID-19 vaccine first booster campaign on August 11, 2021, and a second booster campaign on February 14, 2022, prioritizing individuals with older age, immunocompromised, and those with comorbidities, including chronic kidney disease, diabetes, cardiovascular disease (hypertension, myocardial infarction), stroke, chronic obstructive pulmonary disease, hematological disease (lymphoma, leukemia, myeloma), autoimmune disease (rheumatoid arthritis, juvenile idiopathic arthritis, systemic lupus erythematosus), HIV, and Alzheimer's and other dementias. Our study cohort included adults aged 20 years or older affiliated with the Fondo Nacional de Salud (FONASA), Chile's national public health insurance program.

Supplementary Table S2. Characteristics of the study cohort of adults aged 20 years or older affiliated to FONASA, with laboratory-confirmed COVID-19, unvaccinated and vaccinated individuals who received a BNT162b2 primary series plus a homologous booster (3mRNA) and a second mRNA booster (fourth dose), August 11, 2021, through August 15, 2022*

							Primary vaccination series + booster dose			
			COVID-19		Unvaccinated		First booster		Second booster	
Characteristic	No.	Col.%	No.	Row%	No.	Row%	No.	Row%	No.	Row%
Total	1,020,835	100.0	93,846	9.2	757,726	74.2	22,872	2.2	237,062	23.2
**Sex**										
Female	545,593	53.0	59,837	11.0	361,108	66.2	14,851	2.7	167,753	30.8
Male	475,242	47.0	34,009	7.2	396,618	83.5	8,021	1.7	69,309	14.6
**Region of residence**										
Arica	18,951	1.9	2,672	14.0	12,203	64.4	469	2.5	6,213	32.8
Tarapacá	26,160	2.6	1,640	6.3	18,168	69.5	659	2.5	7,224	27.6
Antofagasta	38,123	3.7	2,722	7.1	25,468	66.8	883	2.3	11,658	30.6
Atacama	18,214	1.8	2,249	12.0	11,565	63.5	544	3.0	6,030	33.1
Coquimbo	54,071	5.3	6,779	13.0	32,102	59.4	1,838	3.4	19,847	36.7
Valparaíso	116,467	11.0	10,285	8.8	93,877	80.6	2,211	1.9	20,090	17.3
Metropolitana	367,390	36.0	23,514	6.4	304,314	82.8	5,212	1.4	57,087	15.5
LB O'Higgins	49,578	4.9	5,102	10.0	32,769	66.1	1,135	2.3	15,507	31.3
Maule	57,196	5.6	7,320	13.0	39,533	69.1	1,653	2.9	15,790	27.6
Ñuble	28,357	2.8	4,686	17.0	16,588	58.5	858	3.0	10,813	38.1
Biobío	83,982	8.2	9,660	12.0	52,439	62.4	2,488	3.0	28,771	34.3
Araucanía	63,625	6.2	7,154	11.0	47,930	75.3	1,646	2.6	13,827	21.7
Los Ríos	24,645	2.4	2,660	11.0	17,231	69.9	785	3.2	6,494	26.4
Los Lagos	57,294	5.6	5,283	9.2	42,400	74.0	1,993	3.5	12,622	22.0
Aysén	7,223	0.7	1,127	16.0	4,100	56.8	298	4.1	2,791	38.6
Magallanes	9,559	0.9	993	10.0	7,039	73.6	200	2.1	2,298	24.0
**Age group†**										
20-29	173,190	17.0	17,972	10.0	147,632	85.2	2,407	1.4	22,868	13.2
30-39	223,971	22.0	23,082	10.0	175,679	78.4	4,427	2.0	43,305	19.3
40-49	175,781	17.0	17,705	10.0	123,358	70.2	4,029	2.3	47,936	27.3
50-59	205,860	20.0	19,384	9.4	103,081	50.1	7,407	3.6	94,493	45.9
60-69	104,497	10.0	6,738	6.4	76,780	73.5	3,222	3.1	23,968	22.9
70-79	55,622	5.4	3,035	5.5	51,925	93.4	799	1.4	2,653	4.8
80 or more	49,219	4.8	3,705	7.5	47,756	97.0	420	0.9	837	1.7
**Comorbidities**										
None	756,380	74.0	62,624	8.3	600,004	79.3	13,101	1.7	141,479	18.7
≥ 1	264,455	26.0	31,222	12.0	157,722	59.6	9,771	3.7	95,583	36.1
**Nationality**										
Chilean	861,707	84.0	90,485	11.0	605,160	70.2	22,191	2.6	231,401	26.9
Non-Chilean	159,128	16.0	3,361	2.1	152,566	95.9	681	0.4	5,661	3.6

Notes. ***COVID-19 denotes coronavirus disease 2019. †The analysis uses age in years; the table includes age groups to show the age distribution in the cohort. The Ministry of Health launched a COVID-19 vaccine first booster campaign in August 11, 2021, and a second booster campaign on February 14, 2022, prioritizing individuals with older age, immunocompromised, and those with comorbidities, including chronic kidney disease, diabetes, cardiovascular disease (hypertension, myocardial infarction), stroke, chronic obstructive pulmonary disease, hematological disease (lymphoma, leukemia, myeloma), autoimmune disease (rheumatoid arthritis, juvenile idiopathic arthritis, systemic lupus erythematosus), HIV, and Alzheimer's and other dementias. Our study cohort included adults aged 20 years or older affiliated with the Fondo Nacional de Salud (FONASA), Chile's national public health insurance program.

Supplementary Table S3. Characteristics of the study cohort of adults aged 20 years or older affiliated to FONASA, with laboratory-confirmed COVID-19, unvaccinated and vaccinated individuals who received an inactivated SARS-CoV-2 vaccine Sinovac primary series plus a heterologous mRNA booster (CC+mRNA) and a second mRNA booster (fourth dose), August 11, 2021, through August 15, 2022*

							Primary vaccination series + booster dose			
			COVID-19		Unvaccinated		First booster		Second booster	
Characteristic	No.	Col.%	No.	Row%	No.	Row%	No.	Row%	No.	Row%
Total	1,837,534	100.0	203,574	11.1	757,726	41.2	174,465	9.5	841,122	45.8
**Sex**										
Female	1,051,890	57.0	135,035	13.0	361,108	34.3	104,195	9.9	549,600	52.3
Male	785,644	43.0	68,539	8.7	396,618	50.5	70,270	8.9	291,522	37.1
**Region of residence**										
Arica	25,527	1.4	3,226	13.0	12,203	47.8	1,796	7.0	10,872	42.6
Tarapacá	35,481	1.9	3,199	9.0	18,168	51.2	2,498	7.0	14,062	39.6
Antofagasta	50,168	2.7	3,466	6.9	25,468	50.8	3,655	7.3	19,794	39.5
Atacama	29,341	1.6	3,526	12.0	11,565	39.4	2,776	9.5	14,182	48.3
Coquimbo	79,957	4.4	8,772	11.0	32,102	40.2	7,405	9.3	37,615	47.0
Valparaíso	218,714	12.0	24,235	11.0	93,877	42.9	19,676	9.0	97,084	44.4
Metropolitana	650,575	35.0	62,066	9.5	304,314	46.8	52,508	8.1	274,048	42.1
LB O'Higgins	98,558	5.4	9,197	9.3	32,769	33.3	10,649	10.8	51,302	52.1
Maule	118,720	6.5	17,619	15.0	39,533	33.3	13,398	11.3	61,235	51.6
Ñuble	60,091	3.3	8,723	15.0	16,588	27.6	7,352	12.2	33,826	56.3
Biobío	171,795	9.3	21,692	13.0	52,439	30.5	18,887	11.0	94,628	55.1
Araucanía	117,098	6.4	15,840	14.0	47,930	40.9	13,174	11.3	50,302	43.0
Los Ríos	46,146	2.5	6,391	14.0	17,231	37.3	4,914	10.7	22,436	48.6
Los Lagos	104,237	5.7	11,406	11.0	42,400	40.7	12,859	12.3	43,502	41.7
Aysén	12,157	0.7	1,752	14.0	4,100	33.7	1,297	10.7	6,459	53.1
Magallanes	18,969	1.0	2,464	13.0	7,039	37.1	1,621	8.5	9,775	51.5
**Age group†**										
20-29	264,666	14.0	36,901	14.0	147,632	55.8	14,055	5.3	101,053	38.2
30-39	329,594	18.0	45,857	14.0	175,679	53.3	17,396	5.3	133,908	40.6
40-49	307,546	17.0	38,950	13.0	123,358	40.1	16,058	5.2	166,015	54.0
50-59	294,152	16.0	31,134	11.0	103,081	35.0	20,266	6.9	167,200	56.8
60-69	265,330	14.0	21,099	8.0	76,780	28.9	40,677	15.3	134,162	50.6
70-79	194,195	11.0	14,650	7.5	51,925	26.7	34,671	17.9	90,792	46.8
80 or more	145,789	7.9	12,292	8.4	47,756	32.8	30,530	20.9	44,158	30.3
**Comorbidities**										
None	1,158,371	63.0	123,534	11.0	600,004	51.8	77,459	6.7	458,404	39.6
≥ 1	679,163	37.0	80,040	12.0	157,722	23.2	97,006	14.3	382,718	56.4
**Nationality**										
Chilean	1,658,844	90.0	197,864	12.0	605,160	36.5	170,566	10.3	821,027	49.5
Non-Chilean	178,690	9.7	5,710	3.2	152,566	85.4	3,899	2.2	20,095	11.3

**Notes. ***COVID-19 denotes coronavirus disease 2019. †The analysis uses age in years; the table includes age groups to show the age distribution in the cohort. The Ministry of Health launched a COVID-19 vaccine first booster campaign in August 11, 2021, and a second booster campaign on February 14, 2022, prioritizing individuals with older age, immunocompromised, and those with comorbidities, including chronic kidney disease, diabetes, cardiovascular disease (hypertension, myocardial infarction), stroke, chronic obstructive pulmonary disease, hematological disease (lymphoma, leukemia, myeloma), autoimmune disease (rheumatoid arthritis, juvenile idiopathic arthritis, systemic lupus erythematosus), HIV, and Alzheimer's and other dementias. Our study cohort included adults aged 20 years or older affiliated with the Fondo Nacional de Salud (FONASA), Chile's national public health insurance program.

Supplementary Table S4. Characteristics of the study cohort of adults aged 20 years or older affiliated to FONASA, with laboratory-confirmed COVID-19, unvaccinated and vaccinated individuals who received an inactivated SARS-CoV-2 vaccine Sinovac primary series plus a homologous booster (CCC) and a second booster (fourth dose) based on mRNA vaccine, August 11, 2021, through August 15, 2022*

							Primary vaccination series + booster dose			
			COVID-19		Unvaccinated		First booster		Second booster	
Characteristic	No.	Col.%	No.	Row%	No.	Row%	No.	Row%	No.	Row%
Total	975,150	100.0	74,867	7.7	757,726	77.7	16,038	1.6	137,165	14.1
**Sex**										
Female	491,062	50.0	41,152	8.4	361,108	73.5	9,498	1.9	83,469	17.0
Male	484,088	50.0	33,715	7.0	396,618	81.9	6,540	1.4	53,696	11.1
**Region of residence**										
Arica	13,347	1.4	1,062	8.0	12,203	91.4	100	0.7	388	2.9
Tarapacá	21,065	2.2	1,176	5.6	18,168	86.3	187	0.9	1,957	9.3
Antofagasta	29,004	3.0	1,274	4.4	25,468	87.8	178	0.6	2,107	7.3
Atacama	12,536	1.3	1,059	8.4	11,565	92.3	18	0.1	135	1.1
Coquimbo	36,431	3.7	2,980	8.2	32,102	88.1	164	0.5	1,330	3.7
Valparaíso	115,671	12.0	8,834	7.6	93,877	81.2	1,380	1.2	12,337	10.7
Metropolitana	396,899	41.0	25,783	6.5	304,314	76.7	6,808	1.7	66,072	16.7
LB O’Higgins	44,569	4.6	3,070	6.9	32,769	73.5	653	1.5	7,309	16.4
Maule	52,388	5.4	5,570	11.0	39,533	75.5	783	1.5	7,518	14.4
Ñuble	24,361	2.5	2,571	11.0	16,588	68.1	566	2.3	4,882	20.0
Biobío	73,401	7.5	7,261	9.9	52,439	71.4	1,710	2.3	13,411	18.3
Araucanía	60,915	6.2	5,739	9.4	47,930	78.7	1,035	1.7	6,258	10.3
Los Ríos	22,690	2.3	2,375	10.0	17,231	75.9	531	2.3	3,363	14.8
Los Lagos	57,849	5.9	4,778	8.3	42,400	73.3	1,678	2.9	8,295	14.3
Aysén	4,661	0.5	514	11.0	4,100	88.0	28	0.6	232	5.0
Magallanes	9,363	1.0	821	8.8	7,039	75.2	219	2.3	1,571	16.8
**Age group†**										
20-29	150,010	15.0	12,126	8.1	147,632	98.4	45	0.0	407	0.3
30-39	179,051	18.0	12,520	7.0	175,679	98.1	104	0.1	657	0.4
40-49	126,729	13.0	8,203	6.5	123,358	97.3	125	0.1	1,131	0.9
50-59	120,598	12.0	8,885	7.4	103,081	85.5	868	0.7	13,044	10.8
60-69	148,144	15.0	12,301	8.3	76,780	51.8	3,669	2.5	53,984	36.4
70-79	117,960	12.0	9,728	8.2	51,925	44.0	4,794	4.1	44,434	37.7
80 or more	101,023	10.0	9,011	8.9	47,756	47.3	6,428	6.4	23,494	23.3
**Comorbidities**										
None	661,498	68.0	40,842	6.2	600,004	90.7	3,543	0.5	35,447	5.4
≥ 1	313,652	32.0	34,025	11.0	157,722	50.3	12,495	4.0	101,718	32.4
**Nationality**										
Chilean	819,034	84.0	72,234	8.8	605,160	73.9	15,869	1.9	135,914	16.6
Non-Chilean	156,116	16.0	2,633	1.7	152,566	97.7	169	0.1	1,251	0.8

Notes. *COVID-19 denotes coronavirus disease 2019. †The analysis uses age in years; the table includes age groups to show the age distribution in the cohort. The Ministry of Health launched a COVID-19 vaccine first booster campaign in August 11, 2021, and a second booster campaign on February 14, 2022, prioritizing individuals with older age, immunocompromised, and those with comorbidities, including chronic kidney disease, diabetes, cardiovascular disease (hypertension, myocardial infarction), stroke, chronic obstructive pulmonary disease, hematological disease (lymphoma, leukemia, myeloma), autoimmune disease (rheumatoid arthritis, juvenile idiopathic arthritis, systemic lupus erythematosus), HIV, and Alzheimer's and other dementias. Our study cohort included adults aged 20 years or older affiliated with the Fondo Nacional de Salud (FONASA), Chile's national public health insurance program.

Supplementary Table S5. Characteristics of the study cohort of adults aged 20 years or older affiliated to FONASA, with laboratory-confirmed COVID-19, unvaccinated and vaccinated individuals who received an inactivated SARS-CoV-2 vaccine Sinovac primary series plus a heterologous viral-vectored ChAdOx1 booster (CCA) and a second booster (fourth dose) based on mRNA vaccine, August 11, 2021, through August 15, 2022*

							Primary vaccination series + booster dose			
			COVID-19		Unvaccinated		First booster		Second booster	
Characteristic	No.	Col.%	No.	Row%	No.	Row%	No.	Row%	No.	Row%
Total	2,307,795	100.0	190,413	8.3	757,726	32.8	91,424	4.0	1,394,424	60.4
**Sex**										
Female	1,248,187	54.0	108,873	8.7	361,108	28.9	50,199	4.0	799,893	64.1
Male	1,059,608	46.0	81,540	7.7	396,618	37.4	41,225	3.9	594,531	56.1
**Region of residence**										
Arica	25,717	1.1	2,705	11.0	12,203	47.5	640	2.5	12,218	47.5
Tarapacá	35,330	1.5	2,332	6.6	18,168	51.4	1,003	2.8	15,406	43.6
Antofagasta	61,050	2.6	3,293	5.4	25,468	41.7	1,920	3.1	32,411	53.1
Atacama	34,927	1.5	3,336	9.6	11,565	33.1	1,231	3.5	21,313	61.0
Coquimbo	104,881	4.5	8,564	8.2	32,102	30.6	4,190	4.0	65,754	62.7
Valparaíso	277,631	12.0	23,151	8.3	93,877	33.8	12,401	4.5	163,276	58.8
Metropolitana	903,611	39.0	68,027	7.5	304,314	33.7	31,230	3.5	548,362	60.7
LB O'Higgins	120,556	5.2	7,985	6.6	32,769	27.2	4,768	4.0	79,181	65.7
Maule	148,286	6.4	15,687	11.0	39,533	26.7	5,691	3.8	98,508	66.4
Ñuble	70,699	3.1	7,230	10.0	16,588	23.5	2,936	4.2	48,850	69.1
Biobío	194,864	8.4	18,200	9.3	52,439	26.9	7,630	3.9	128,954	66.2
Araucanía	143,993	6.2	13,899	9.7	47,930	33.3	8,222	5.7	82,149	57.1
Los Ríos	47,122	2.0	4,658	9.9	17,231	36.6	2,203	4.7	26,123	55.4
Los Lagos	110,156	4.8	8,363	7.6	42,400	38.5	6,373	5.8	55,907	50.8
Aysén	9,259	0.4	990	11.0	4,100	44.3	376	4.1	4,482	48.4
Magallanes	19,713	0.9	1,993	10.0	7,039	35.7	610	3.1	11,530	58.5
**Age group†**										
20-29	149,580	6.5	11,994	8.0	147,632	98.7	1	0.0	21	0.0
30-39	178,323	7.7	12,318	6.9	175,679	98.5	-	-	33	0.0
40-49	126,215	5.5	8,035	6.4	123,358	97.7	37	0.0	705	0.6
50-59	266,597	12.0	27,169	10.0	103,081	38.7	6,845	2.6	153,066	57.4
60-69	785,242	34.0	69,103	8.8	76,780	9.8	32,919	4.2	661,832	84.3
70-79	541,493	23.0	41,268	7.6	51,925	9.6	27,509	5.1	445,252	82.2
80 or more	228,728	9.9	18,435	8.1	47,756	20.9	24,113	10.5	133,514	58.4
**Comorbidities**										
None	1,182,133	51.0	81,794	6.9	600,004	50.8	31,925	2.7	527,700	44.6
≥ 1	1,125,662	49.0	108,619	9.6	157,722	14.0	59,499	5.3	866,724	77.0
**Nationality**										
Chilean	2,134,038	92.0	186,280	8.7	605,160	28.4	89,971	4.2	1,376,816	64.5
Non-Chilean	173,757	7.5	4,133	2.4	152,566	87.8	1,453	0.8	17,608	10.1

**Notes. ***COVID-19 denotes coronavirus disease 2019. †The analysis uses age in years; the table includes age groups to show the age distribution in the cohort. The Ministry of Health launched a COVID-19 vaccine first booster campaign in August 11, 2021, and a second booster campaign on February 14, 2022, prioritizing individuals with older age, immunocompromised, and those with comorbidities, including chronic kidney disease, diabetes, cardiovascular disease (hypertension, myocardial infarction), stroke, chronic obstructive pulmonary disease, hematological disease (lymphoma, leukemia, myeloma), autoimmune disease (rheumatoid arthritis, juvenile idiopathic arthritis, systemic lupus erythematosus), HIV, and Alzheimer's and other dementias. Our study cohort included adults aged 20 years or older affiliated with the Fondo Nacional de Salud (FONASA), Chile's national public health insurance program.

Supplementary Table S6. Weekly effectiveness against ICU admissions of mRNA-based second vaccine boosters for individuals with four different three-dose background regimes: (i) BNT162b2 primary series plus a homologous booster, a CoronaVac primary series plus (ii) mRNA booster, (iii) homologous booster, or (iv) ChAdOx-1 booster, compared to unvaccinated individuals among adults aged 20 years and older, March 7, 2022, through August 15, 2022*

Report date	Overall		3mRNA+mRNA		CC+mRNA+mRNA		CCC+mRNA		CCA+mRNA	
	VE (%)	95% CI	VE (%)	95% CI	VE (%)	95% CI	VE (%)	95% CI	VE (%)	95% CI
March 7	96.8	(86.8-99.2)	100		100.0		100.0		95.6	(81.9-99)
March 14	94.1	(88.3-97.0)	100		93.0	(49.6-99.0)	88.0	(13.6-98.3)	94.7	(87.7-97.7)
March 21	96.3	(92.9-98.1)	100		92.0	(66.6-98.0)	93.6	(53.6-99.1)	96.9	(92.9-98.7)
March 28	95.9	(92.9-97.6)	100		90.0	(68.6-96.9)	95.0	(64.0-99.3)	96.3	(92.7-98.1)
April 4	95.7	(93.1-97.3)	100		87.0	(67.2-94.6)	92.2	(68.1-98.1)	96.4	(93.2-98.1)
April 11	95.5	(93.1-97.1)	100		83.0	(63.6-92.2)	92.9	(71.2-98.3)	96.1	(93.0-97.8)
April 18	96.0	(93.8-97.4)	100		85.0	(67.4-93.0)	93.7	(74.5-98.5)	96.5	(93.7-98.0)
April 25	96.0	(94.0-97.4)	100		86.0	(69.4-93.4)	91.2	(72.2-97.2)	96.6	(94.0-98.1)
May 2	95.7	(93.6-97.1)	100		86.0	(70.4-93.6)	88.7	(69.3-95.8)	96.3	(93.6-97.8)
May 9	95.5	(93.5-96.9)	100		88.0	(73.3-94.2)	89.6	(71.8-96.2)	95.7	(92.9-97.3)
May 16	94.9	(92.8-96.4)	100		88.0	(74.6-94.5)	85.2	(66.3-93.5)	95.0	(92.2-96.8)
May 23	94.5	(92.4-96.0)	100		84.0	(69.9-91.8)	85.9	(67.8-93.8)	94.2	(91.2-96.2)
May 30	94.2	(92.1-95.7)	85.5	(0.0-98.0)	85.0	(71.9-91.9)	87.2	(70.9-94.4)	93.5	(90.5-95.5)
June 6	93.1	(91.0-94.7)	70.8	(5.6-91.0)	84.0	(72.0-90.8)	88.7	(74.2-95.0)	92.4	(89.4-94.6)
June 13	92.3	(90.2-93.9)	74.2	(28.4-90.7)	80.0	(68.8-87.5)	82.5	(67.5-90.6)	92.1	(89.2-94.2)
June 20	91.8	(89.8-93.4)	75.9	(39.8-90.3)	80.0	(69.4-86.8)	77.7	(62.5-86.8)	91.4	(88.6-93.6)
June 27	90.8	(88.7-92.5)	75.8	(44.0-89.5)	78.0	(68.2-85.3)	75.9	(61.0-85.1)	89.8	(86.7-92.2)
July 4	90.7	(88.8-92.3)	77.2	(50.4-89.5)	78.0	(68.2-84.4)	78.6	(65.4-86.7)	89.7	(86.7-92.0)
July 11	89.7	(87.7-91.4)	79.9	(56.4-90.7)	78.0	(68.6-84.1)	78.0	(65.2-86.0)	87.9	(84.8-90.4)
July 18	89.3	(87.3-91.0)	75.8	(51.8-87.8)	78.0	(69.0-84.1)	74.8	(61.5-83.5)	87.1	(83.9-89.7)
July 25	88.5	(86.4-90.2)	72.4	(48.3-85.3)	77.0	(68.3-83.4)	72.9	(59.4-81.9)	86.0	(82.6-88.7)
August 01	88.5	(86.4-90.2)	74.2	(53.0-85.9)	77.0	(69.1-83.3)	74.3	(61.9-82.7)	86.3	(83.1-88.9)
August 08	88.3	(86.3-90.0)	75.0	(55.4-86.0)	77.0	(68.7-82.6)	74.4	(62.5-82.5)	86.7	(83.6-89.2)
August 15	88.2	(86.2-89.9)	74.2	(54.8-85.2)	77.0	(69.7-83.0)	75.0	(63.4-82.9)	86.3	(83.2-88.8)

*****COVID-19 denotes coronavirus disease 2019. The Ministry of Health launched a COVID-19 vaccine first booster campaign in August 11, 2021, and a second booster campaign on February 14, 2022. The table shows the estimated vaccine effectiveness of mRNA-based second vaccine boosters for individuals with four different three-dose background regimes: i) BNT162b2 primary series plus a homologous booster (3mRNA), a CoronaVac primary series plus (ii) mRNA booster (CC+mRNA), iii) homologous booster (CCC), or (iv) ChAdOx-1 booster (CCA), compared to unvaccinated individuals. Estimates were adjusted for time-varying vaccination exposure and clinical, demographic, and socioeconomic confounders at baseline (Supplementary Tables S1-S5).

Supplementary Table S7. Weekly effectiveness against confirmed death of mRNA-based second vaccine boosters for individuals with four different three-dose background regimes: (i) BNT162b2 primary series plus a homologous booster, a CoronaVac primary series plus (ii) mRNA booster, (iii) homologous booster, or (iv) ChAdOx-1 booster, compared to unvaccinated individuals among adults aged 20 years and older, March 7, 2022, through August 15, 2022*

Report date	Overall		3mRNA+mRNA		CC+mRNA+mRNA		CCC+mRNA		CCA+mRNA	
	VE (%)	95% CI	VE (%)	95% CI	VE (%)	95% CI	VE (%)	95% CI	VE (%)	95% CI
March 7	96.5	(93.0-98.3)	100.0		95.0	(64.3-99.3)	79.6	(17.2-95.0)	96.5	(91.3-98.6)
March 14	96.6	(94.6-97.9)	100.0		98.0	(86.3-99.7)	89.4	(66.7-96.6)	96.2	(93.4-97.8)
March 21	96.9	(95.4-97.9)	100.0		98.0	(90.1-99.4)	93.6	(80.1-98.0)	96.5	(94.4-97.8)
March 28	96.7	(95.3-97.7)	100.0		97.0	(91.1-99.1)	93.8	(83.2-97.7)	96.5	(94.7-97.6)
April 4	96.5	(95.3-97.4)	100.0		96.0	(90.6-98.4)	91.3	(81.6-95.9)	96.4	(94.8-97.5)
April 11	96.6	(95.5-97.5)	100.0		97.0	(91.6-98.6)	92.4	(83.8-96.4)	96.5	(95.0-97.5)
April 18	96.6	(95.5-97.5)	100.0		96.0	(91.3-98.3)	92.9	(85.0-96.7)	96.4	(95.0-97.4)
April 25	96.4	(95.3-97.3)	100.0		95.0	(90.2-97.6)	90.6	(82.3-95.0)	96.4	(95.0-97.4)
May 2	96.2	(95.1-97.1)	100.0		95.0	(90.5-97.7)	90.0	(81.8-94.5)	96.2	(94.8-97.2)
May 9	95.8	(94.7-96.7)	100.0		96.0	(91.2-97.8)	89.1	(80.9-93.7)	95.7	(94.3-96.8)
May 16	95.5	(94.4-96.4)	100.0		95.0	(90.9-97.6)	88.8	(80.9-93.5)	95.2	(93.7-96.4)
May 23	95.2	(94.1-96.1)	100.0		94.0	(89.2-96.4)	88.2	(80.4-92.8)	95.0	(93.5-96.1)
May 30	95.0	(93.9-95.9)	100.0		93.0	(87.9-95.5)	87.7	(80.2-92.4)	94.8	(93.3-95.9)
June 6	94.1	(92.9-95.0)	100.0		90.0	(84.8-93.1)	84.8	(77.1-89.9)	94.0	(92.5-95.2)
June 13	93.4	(92.3-94.4)	100.0		87.0	(82.0-90.8)	85.1	(78.0-90.0)	93.2	(91.7-94.5)
June 20	92.9	(91.8-93.9)	100.0		86.0	(81.0-89.5)	82.8	(75.8-87.8)	92.8	(91.3-94.1)
June 27	92.5	(91.4-93.4)	100.0		86.0	(81.8-89.6)	81.0	(74.0-86.0)	92.2	(90.7-93.5)
July 4	92.1	(91.0-93.0)	90.1	(73.3-96.3)	84.0	(79.8-87.6)	81.4	(75.0-86.1)	91.9	(90.5-93.2)
July 11	91.5	(90.4-92.4)	90.1	(76.0-95.9)	83.0	(79.3-86.8)	80.1	(74.0-84.8)	91.5	(90.0-92.7)
July 18	91.0	(89.9-92.0)	89.2	(75.7-95.2)	83.0	(78.7-86.1)	78.2	(72.0-83.1)	91.0	(89.6-92.3)
July 25	90.6	(89.6-91.6)	88.6	(75.7-94.6)	82.0	(77.9-85.3)	78.5	(72.5-83.2)	90.6	(89.1-91.8)
August 01	90.5	(89.5-91.5)	86.7	(74.2-93.2)	81.0	(77.0-84.4)	79.3	(73.6-83.7)	90.7	(89.3-91.9)
August 08	90.6	(89.5-91.5)	87.6	(75.8-93.6)	81.0	(77.3-84.4)	79.7	(74.1-84.0)	90.9	(89.5-92.1)
August 15	90.5	(89.4-91.4)	87.7	(76.1-93.7)	81.0	(76.8-84.0)	79.3	(73.8-83.7)	90.8	(89.4-92.0)

*COVID-19 denotes coronavirus disease 2019. The Ministry of Health launched a COVID-19 vaccine first booster campaign in August 11, 2021, and a second booster campaign on February 14, 2022. The table shows the estimated vaccine effectiveness of mRNA-based second vaccine boosters for individuals with four different three-dose background regimes: i) BNT162b2 primary series plus a homologous booster (3mRNA), a CoronaVac primary series plus (ii) mRNA booster (CC+mRNA), iii) homologous booster (CCC), or (iv) ChAdOx-1 booster (CCA), compared to unvaccinated individuals. Estimates were adjusted for time-varying vaccination exposure and clinical, demographic, and socioeconomic confounders at baseline (Supplementary Tables S1-S5).

Supplementary Table S8. Overall weekly vaccine effectiveness against ICU admissions and death for adults >20 years of mRNA-based second vaccine boosters compared to three-dose background regimes in Chile, March 7, 2022, through August 15, 2022

Report date	ICU admissions		Death	
	VE (%)	95% CI	VE (%)	95% CI
March 7	90.9	(61.3-97.9)	93.8	(87.3-96.9)
March 14	78.1	(53.5-89.7)	92.9	(88.4-95.6)
March 21	87.2	(74.2-93.7)	92.9	(89.3-95.3)
March 28	87.5	(77.5-93.0)	91.7	(88.1-94.2)
April 4	85.4	(75.5-91.3)	91.2	(87.8-93.6)
April 11	85.8	(77.1-91.2)	91.0	(87.8-93.4)
April 18	86.8	(78.8-91.8)	90.7	(87.4-93.1)
April 25	85.1	(76.3-90.6)	89.7	(86.3-92.2)
May 2	83.2	(73.8-89.2)	88.4	(84.8-91.2)
May 9	82.4	(73.2-88.5)	86.9	(83.1-89.9)
May 16	77.9	(67.2-85.1)	85.2	(81.1-88.4)
May 23	76.6	(66.0-83.8)	83.8	(79.6-87.1)
May 30	74.0	(63.1-81.6)	82.8	(78.6-86.2)
June 6	68.9	(57.0-77.5)	80.0	(75.6-83.6)
June 13	68.1	(57.0-76.4)	78.7	(74.4-82.3)
June 20	68.8	(58.8-76.4)	77.2	(73.0-80.8)
June 27	67.4	(57.7-74.9)	76.3	(72.2-79.9)
July 4	68.4	(59.6-75.3)	75.5	(71.5-79.0)
July 11	65.3	(56.1-72.6)	73.9	(69.8-77.3)
July 18	65.2	(56.4-72.3)	72.8	(68.8-76.3)
July 25	62.8	(53.8-70.1)	71.6	(67.6-75.1)
August 01	62.0	(52.9-69.3)	70.8	(66.8-74.4)
August 08	61.1	(52.1-68.5)	70.6	(66.6-74.2)
August 15	60.9	(51.9-68.2)	70.1	(66.1-73.7)

*****COVID-19 denotes coronavirus disease 2019. The Ministry of Health launched a COVID-19 vaccine first booster campaign in August 11, 2021, and a second booster campaign on February 14, 2022. Estimates were adjusted for time-varying vaccination exposure and clinical, demographic, and socioeconomic confounders at baseline (Supplementary Tables S1-S5)

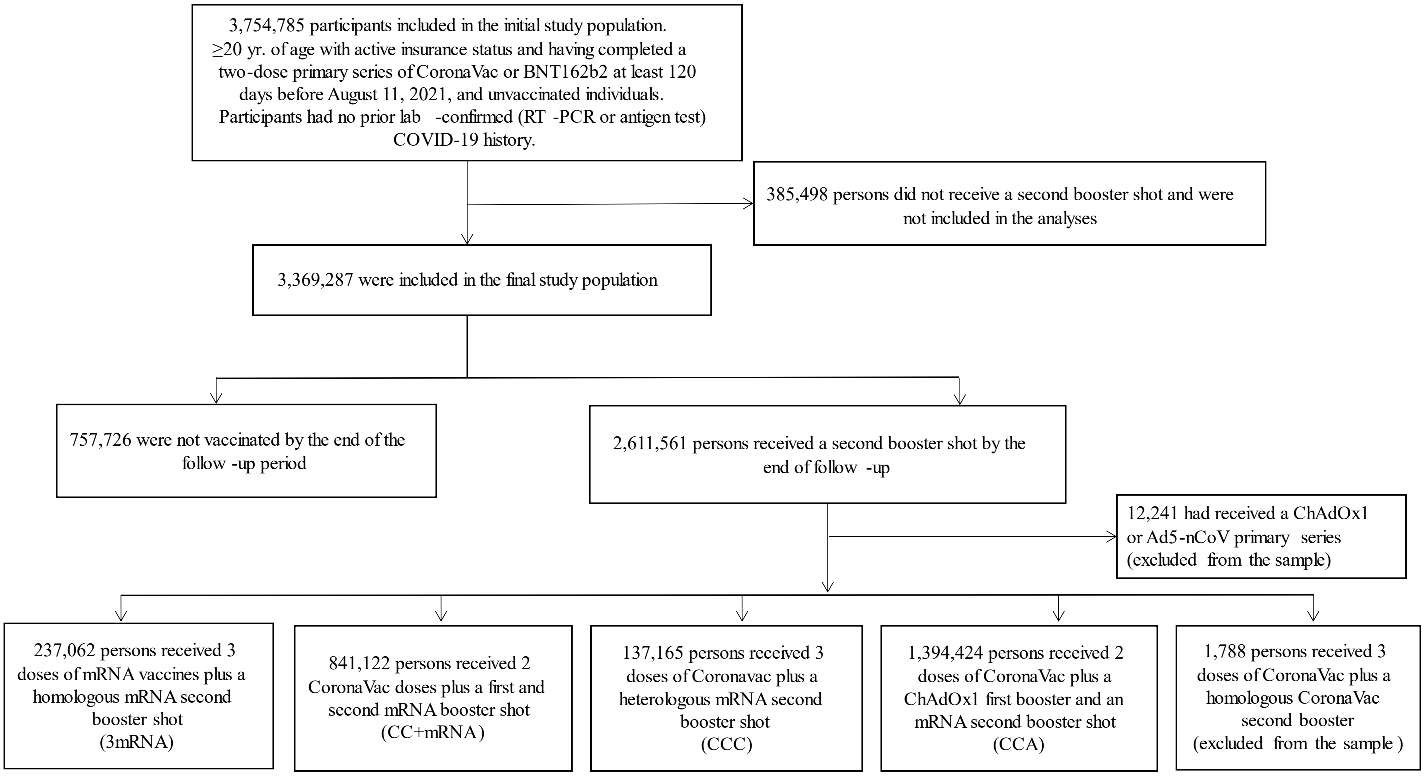

Supplementary Fig.S1. Study participants and cohort eligibility. Participants were adults aged ≥20 years affiliated with the Fondo Nacional de Salud (FONASA), the public national healthcare system in Chile, who completed a Coronavac or BNT162b2's two-dose primary series at least 120 days before the beginning of the follow-up on August 11, 2021, when the first-booster campaign was launched and unvaccinated individuals. We excluded individuals with confirmed COVID-19 according to reverse-transcription polymerase-chain-reaction assay for SARS-CoV-2 or antigen test reported before August 11, 2021. We excluded individuals who received three doses of CoronaVac plus a homologous CoronaVac second booster due to the small sample size (n=1,788) and individuals with a second vaccine booster who had received a primary series of ChAdOx1 or Ad5-nCoV (n=12,241).

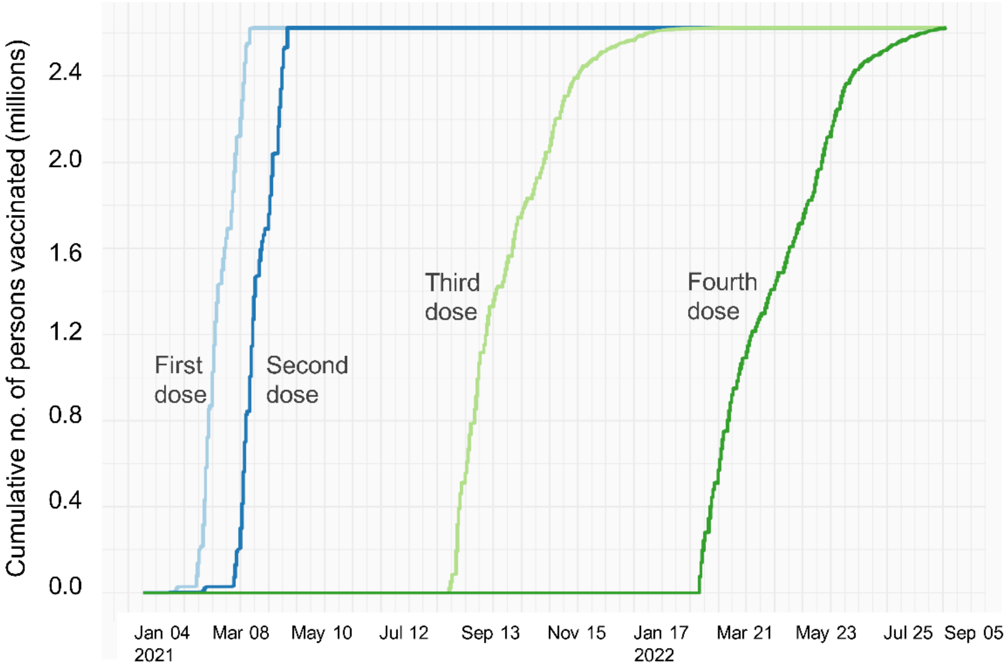

Supplementary Fig.S2. Vaccination rollout for adults aged 20 years and older who received a primary COVID-19 vaccination schedule and two booster doses. The Ministry of Health launched a COVID-19 vaccine first booster campaign on August 11, 2021, and the second booster on February 14, 2022. Our study cohort included adults aged 20 years or older affiliated with the Fondo Nacional de Salud (FONASA), Chile's national public health insurance program.

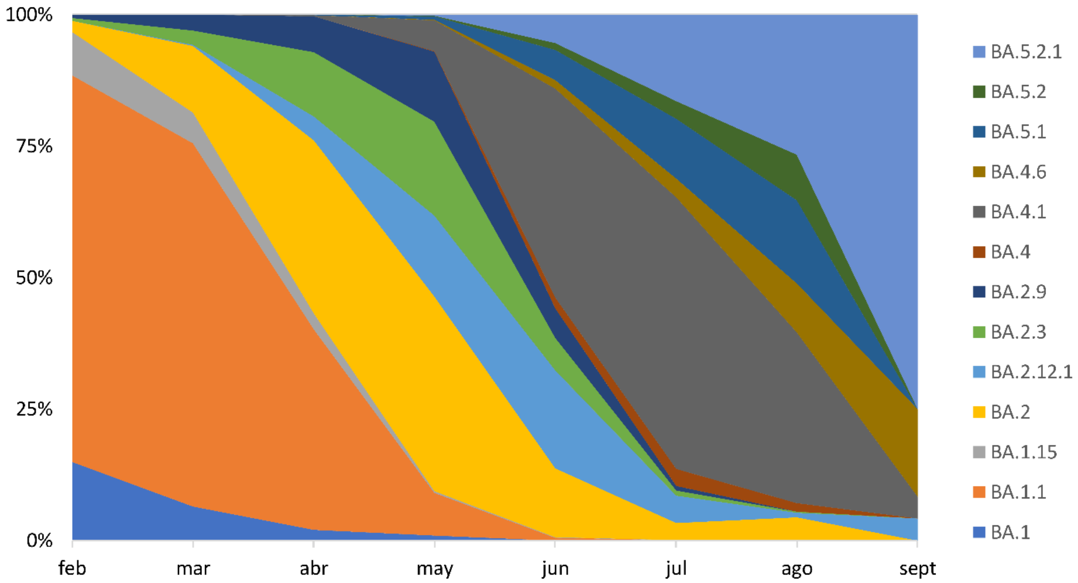

Supplementary Fig.S3. Distribution of the predominant SARS-CoV-2 lineages in Chile between February and September, 2022, based on data shared on the GISAID platform. A total of 12,578 SARS-CoV genomes were available at the GISAID EpiCov repository. The Ministry of Health monitors respiratory viruses, including SARS-CoV-2, using genomic surveillance in sentinel centers. Surveillance efforts related to the pandemic include sequencing a non-probabilistic sample of SARS-CoV-2 RT-PCR positive samples retrieved from travelers entering the country, severe COVID-19 cases, and community surveillance. During the study period, Omicron was the only variant of concern observed. The dynamics of the sub-lineages followed consecutive waves of BA.1, BA.2, BA.2.12.1, BA.4, and BA.5.

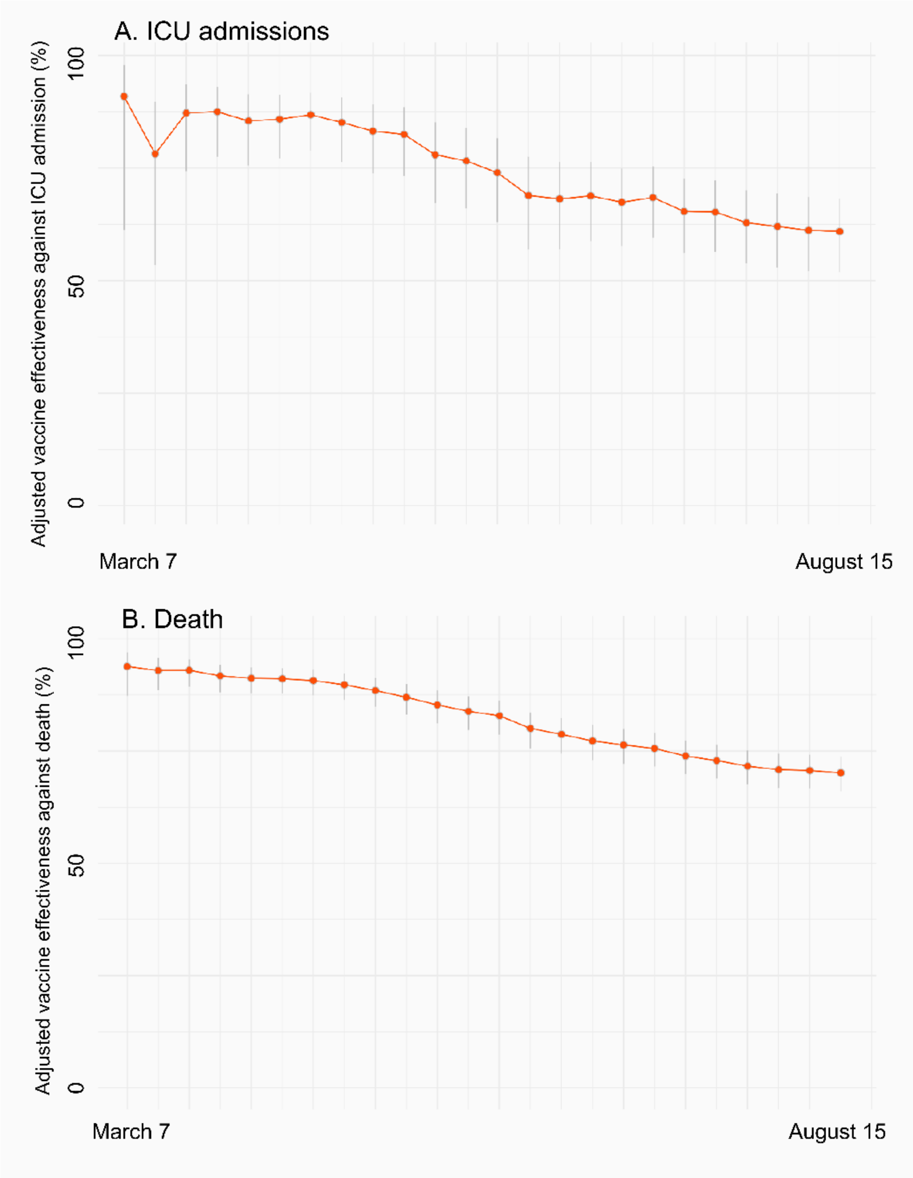

Supplementary Fig.S4. Overall weekly vaccine effectiveness against COVID-19-related (A) ICU admissions and (B) death for adults >20 years of mRNA-based second vaccine boosters compared to three-dose background regimes in Chile, March 7, 2022, through August 15, 2022. Vaccine effectiveness estimates are presented as point estimates with standard 95% Wald confidence intervals. The corresponding numerical values are shown in Supplementary Table S8.
