# Supplementary material for: Effectiveness of the second COVID-19 booster against Omicron: a large-scale cohort study in Chile
Source: Nat Commun. 2023 Oct 27;14:6836. doi: 10.1038/s41467-023-41942-y (PMC10603055; doi:10.1038/s41467-023-41942-y)
Supplement: Supplementary file 3 — Reporting Summary [file 41467_2023_41942_MOESM3_ESM.pdf]

nature portfolio

Corresponding author(s): Rafael Araos

Last updated by author(s): Aug 23, 2023

Reporting Summary

Nature Portfolio wishes to improve the reproducibility of the work that we publish. This form provides structure for consistency and transparency in reporting. For further information on Nature Portfolio policies, see our Editorial Policies and the Editorial Policy Checklist.

Statistics

For all statistical analyses, confirm that the following items are present in the figure legend, table legend, main text, or Methods section.

n/a	Confirmed	
☐	☒	The exact sample size (*n*) for each experimental group/condition, given as a discrete number and unit of measurement
☒	☐	A statement on whether measurements were taken from distinct samples or whether the same sample was measured repeatedly
☐	☒	The statistical test(s) used AND whether they are one- or two-sided<br>*Only common tests should be described solely by name; describe more complex techniques in the Methods section.*
☐	☒	A description of all covariates tested
☐	☒	A description of any assumptions or corrections, such as tests of normality and adjustment for multiple comparisons
☐	☒	A full description of the statistical parameters including central tendency (e.g. means) or other basic estimates (e.g. regression coefficient) AND variation (e.g. standard deviation) or associated estimates of uncertainty (e.g. confidence intervals)
☐	☒	For null hypothesis testing, the test statistic (e.g. *F*, *t*, *r*) with confidence intervals, effect sizes, degrees of freedom and *P* value noted<br>*Give P values as exact values whenever suitable.*
☒	☐	For Bayesian analysis, information on the choice of priors and Markov chain Monte Carlo settings
☒	☐	For hierarchical and complex designs, identification of the appropriate level for tests and full reporting of outcomes
☒	☐	Estimates of effect sizes (e.g. Cohen's *d*, Pearson's *r*), indicating how they were calculated

Our web collection on statistics for biologists contains articles on many of the points above.

Software and code

Policy information about availability of computer code

Data collection	Data were collected as part of the Chilean National surveillance strategy for Covid-19.
Data analysis	We conducted the analysis with the survival package of R, version 4.0.5. (Therneau TM. A Package for Survival Analysis in R. R package version 3.1-12. Computer software] Rochester, MN: Mayo Clinic Retrieved from https://CRAN R-project org/package= survival 2020; R Core Team. R: A language and environment for statistical computing. Vienna: R Foundation for Statistical Computing; 2019)

For manuscripts utilizing custom algorithms or software that are central to the research but not yet described in published literature, software must be made available to editors and reviewers. We strongly encourage code deposition in a community repository (e.g. GitHub). See the Nature Portfolio guidelines for submitting code & software for further information.

Data

Policy information about availability of data

All manuscripts must include a data availability statement. This statement should provide the following information, where applicable:

- Accession codes, unique identifiers, or web links for publicly available datasets
- A description of any restrictions on data availability
- For clinical datasets or third party data, please ensure that the statement adheres to our policy

Owing to data privacy regulations in Chile, this study's individual-level data cannot be shared (Law N19.628). Aggregate data on vaccination, including demographics, and COVID-19 incidence, are publicly available at https://github.com/MinCiencia/Datos-Covid19/.

Code availability

The code for preparing and analyzing data is available upon request from the corresponding author.

Research involving human participants, their data, or biological material

Policy information about studies with human participants or human data. See also policy information about sex, gender (identity/presentation), and sexual orientation and race, ethnicity and racism.

Reporting on sex and gender	Done
Reporting on race, ethnicity, or other socially relevant groupings	Done
Population characteristics	Our final cohort included 3,754,785 adults. Of these, 2,623,802 (69.9) received a second booster shot of BNT162b2 or mRNA-1273 vaccine between February 14, 2022, and August 15, 2022, and 757,726 (20.2%) had not been vaccinated by the end of the follow-up. Vaccination rollout was organized through a publicly available schedule and was free of charge (Extended Fig.2). Notably, there was a high level of SARS-CoV-2 circulation during the rollout, in which the predominant Omicron sub-lineages were BA.2.1.12, BA.4, and BA.5 (Extended Fig.3). Cohort characteristics are described in Extended Data Tables 1-5. The incidence of COVID-19 and the vaccination status at the end of the follow-up differed significantly (P<0.001) by participant's sex, age, comorbidities, nationality, region of residence, and income.
Recruitment	This is an observational study based in the Chilean National Covid-19 surveillance strategy. No formal recruitment was performed.
Ethics oversight	The Comité Ético Científico Clínica Alemana Universidad del Desarrollo, Santiago, Chile, gave ethical approval for this research. The study was considered exempt from informed consent.

Note that full information on the approval of the study protocol must also be provided in the manuscript.

Field-specific reporting

Please select the one below that is the best fit for your research. If you are not sure, read the appropriate sections before making your selection.

- [x] Life sciences
- [ ] Behavioural & social sciences
- [ ] Ecological, evolutionary & environmental sciences

For a reference copy of the document with all sections, see nature.com/documents/nr-reporting-summary-flat.pdf

Life sciences study design

All studies must disclose on these points even when the disclosure is negative.

Sample size	This is a population based analysis of all Chilean residents affiliated with the national health fund, that provides health-coverage to 80% of the country´s population.
Data exclusions	We excluded individuals with confirmed COVID-19 according to reverse-transcription polymerase-chain-reaction assay for SARS-CoV-2 or antigen test reported before August 11, 2021 (Extended Fig.1). We excluded individuals who received three doses of CoronaVac plus a homologous CoronaVac second booster due to the small sample size (n=1,788).
Replication	Does not apply
Randomization	Does not apply
Blinding	Does not apply

Reporting for specific materials, systems and methods

We require information from authors about some types of materials, experimental systems and methods used in many studies. Here, indicate whether each material, system or method listed is relevant to your study. If you are not sure if a list item applies to your research, read the appropriate section before selecting a response.

Materials & experimental systems

n/a	Involved in the study
☒	☐ Antibodies
☒	☐ Eukaryotic cell lines
☒	☐ Palaeontology and archaeology
☒	☐ Animals and other organisms
☒	☐ Clinical data
☒	☐ Dual use research of concern
☒	☐ Plants

Methods

n/a	Involved in the study
☒	☐ ChIP-seq
☒	☐ Flow cytometry
☒	☐ MRI-based neuroimaging
